# Unlocking the tumor-immune microenvironment in osteosarcoma: insights into the immune landscape and mechanisms

**DOI:** 10.3389/fimmu.2024.1394284

**Published:** 2024-09-18

**Authors:** Santhasiri Orrapin, Sutpirat Moonmuang, Sasimol Udomruk, Petlada Yongpitakwattana, Dumnoensun Pruksakorn, Parunya Chaiyawat

**Affiliations:** ^1^ Center of Multidisciplinary Technology for Advanced Medicine (CMUTEAM), Faculty of Medicine, Chiang Mai University, Chiang Mai, Thailand; ^2^ Office of Research Administration, Chiang Mai University, Chiang Mai, Thailand; ^3^ Musculoskeletal Science and Translational Research (MSTR) Center, Faculty of Medicine, Chiang Mai University, Chiang Mai, Thailand; ^4^ Department of Orthopedics, Faculty of Medicine, Chiang Mai University, Chiang Mai, Thailand

**Keywords:** osteosarcoma, tumor-immune microenvironment, immune landscape, mutations, epigenetics, extracellular vesicles

## Abstract

Osteosarcoma has a unique tumor microenvironment (TME), which is characterized as a complex microenvironment comprising of bone cells, immune cells, stromal cells, and heterogeneous vascular structures. These elements are intricately embedded in a mineralized extracellular matrix, setting it apart from other primary TMEs. In a state of normal physiological function, these cell types collaborate in a coordinated manner to maintain the homeostasis of the bone and hematopoietic systems. However, in the pathological condition, i.e., neoplastic malignancies, the tumor-immune microenvironment (TIME) has been shown to promote cancer cells proliferation, migration, apoptosis and drug resistance, as well as immune escape. The intricate and dynamic system of the TIME in osteosarcoma involves crucial roles played by various infiltrating cells, the complement system, and exosomes. This complexity is closely associated with tumor cells evading immune surveillance, experiencing uncontrolled proliferation, and facilitating metastasis. In this review, we elucidate the intricate interplay between diverse cell populations in the osteosarcoma TIME, each contributing uniquely to tumor progression. From chondroblastic and osteoblastic osteosarcoma cells to osteoclasts, stromal cells, and various myeloid and lymphoid cell subsets, the comprehensive single-cell analysis provides a detailed roadmap of the complex osteosarcoma ecosystem. Furthermore, we summarize the mutations, epigenetic mechanisms, and extracellular vesicles that dictate the immunologic landscape and modulate the TIME of osteosarcoma. The perspectives of the clinical implementation of immunotherapy and therapeutic approaches for targeting immune cells are also intensively discussed.

## 1 Introduction

Osteosarcoma is the most common malignant bone tumor, primarily affecting children and adolescents. It is a highly aggressive tumor that commonly results in patient mortality due to metastasis (1). However, this therapeutic approach is limited by metastatic or relapsing osteosarcoma as the current regimen is not entirely curable. Approximate 5-year survival rates are greater than 78% for localized disease, whereas it drops to 20-25% in those who develop chemotherapeutic resistance, metastasis, and recurrence (2–5). Despite therapeutic efforts, there has been minimal improvement in effective treatment options and clinical outcomes for individuals affected by osteosarcoma (6, 7). This challenge arises from multifactorial molecular mechanisms likely involved in drug targets and the development of resistance (8). Consequently, there is an urgent need to consider new therapeutic strategies to effectively eliminate osteosarcoma, especially in the case of metastatic osteosarcoma, and circumvent resistance.

The use of cancer immunotherapy in conjunction with traditional osteosarcoma management has aimed to improve the quality-of-life outcomes in osteosarcoma patients. Strategies involving macrophage modulation, dendritic vaccination, activation of immune-modulating cytokines, immune checkpoint blockade, adoptive cell therapy [such as chimeric antigen receptors (CARs) and T lymphocyte receptors (TCRs)], and combinational immunotherapy have become a focal point in tumor (9, 10). The tumor-immune microenvironment (TIME) plays significant roles in determining the efficacy of cancer immunotherapy (11). This complex ecosystem consists of various components, including malignant cells, endothelial cells, tumor-infiltrating immune cells, and stromal cells, each serving distinct functions. For instance, the types, activity, and quantities of immune cells within the TIME significantly influence the response to cancer immunotherapy. Cytokines and chemokines in the TIME modulate immune cell recruitment, activation, and suppression. Tumor-derived exosomes can carry immunosuppressive molecules that inhibit immune responses. Metabolites produced in TIME can impair immune cell function, support the growth and dissemination of osteosarcoma cells, and contribute to the emergence of drug resistance.

The presented data emphasize the need to study the immune system within the biology of osteosarcoma and gain an understanding of its comprehensive effects, potentially contributing to the successful implementation of novel immunotherapy. In this review, we summarize the immunological landscape existing within osteosarcoma tissue tumors, exploring specific hallmarks modulating the TIME and their clinical implications. We present a future perspective and outlook for novel immunotherapeutic strategies in osteosarcoma, considering the current knowledge centered around the immune microenvironment.

## 2 The immune landscape and tumor-immune microenvironment of osteosarcoma

Osteosarcomas are malignant tumors that develop in the long bones of the limbs, including the femur, tibia, and humerus, and have special molecular and biological characteristics (12). Bone contains a highly specialized immune milieu and immune signaling pathways that are crucial for bone homeostasis. The immune microenvironment within osteosarcoma predominantly consists of T lymphocytes and macrophages, with additional subgroups such as B lymphocytes and mast cells also present. Osteosarcoma has an immunosuppressive TIME characterized by low T-cell infiltration. Overall, osteosarcoma samples have intermediate median immune infiltration scores (ESTIMATE) compared with melanoma and lung cancer, which have high ESTIMATE scores and respond well to immune checkpoint inhibitors (ICIs). Studies of the ICGC and TARGET cohorts show that 10–15% of osteosarcoma samples have high immune infiltration with high ESTIMATE scores. However, osteosarcoma cases with a T-cell presence exhibit low T-cell receptor productive clonality and low activity. T-cell activity reaches a maximum of 0.3 in comparison with normal skin (0.15), with a lack of T-cell clonal diversity and low T-cell clonotypes (<100).

**Figure 1:**
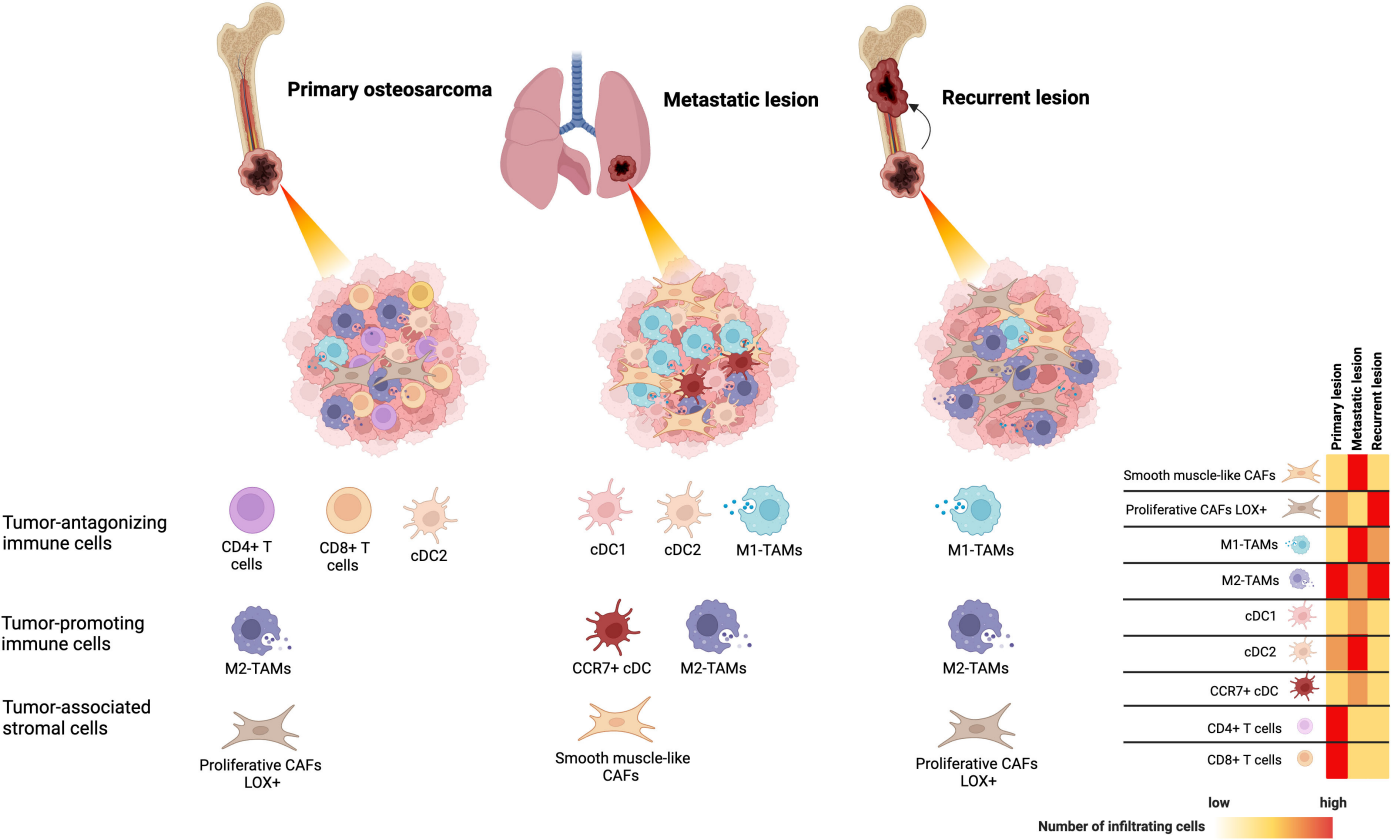
The tumor-immune microenvironment of osteosarcoma identified by single-cell RNA sequencing analysis based on primary, locally recurrent, and metastatic disease.

The association between the TIME and clinical outcomes of osteosarcoma has been widely studied through the analysis of gene expression profiles in immune cells, immunohistochemical examination of archived samples, and single-cell RNA sequencing analysis of osteosarcoma tissues. In osteosarcoma, tumor antagonizing immune cells, particularly activated CD4+ T cells, activated CD8+ T cells, central memory CD4+ and CD8+ T cells, M1 macrophages, natural killer (NK) cells, and tumor-associated neutrophils (TANs) are detected at relatively lower levels in patients with shorter survival rates (13–17). B cells, with controversial roles in cancer, are found at lower levels in patients with poor prognoses (13, 18). Among these infiltrating immune cells, the levels and characteristics of macrophages and T cells are significantly related to key events in the poor prognosis of osteosarcoma, including metastasis and chemoresistance. Decreased M1 macrophage infiltration is observed in metastatic lesions of osteosarcoma and is significantly related to worse overall survival and disease-free survival (19, 20). Increased infiltration of CD4+ T cells, follicular helper (Tfh) cells, and CD8+ T cells is found in patients who respond well to chemotherapeutic treatment (21, 22). Higher levels of memory activated CD4+ T-cell infiltration are associated with better survival outcomes (23). The TIMEs of osteosarcoma are summarized in **Figure 1**.

### 2.1 Monocytes and tumor-associated macrophages

Monocytes play a crucial role in the TIME, acting as a link between the innate and adaptive immune systems during cancer development (24). They exhibit diverse functions in both pro-tumoral and anti-tumoral immunity, such as phagocytosis, lymphocyte recruitment, angiogenesis, and differentiation into TAMs and monocyte-derived dendritic cells (DCs). Two subtypes of monocytes, classical (CD14+CD16-) and non-classical (CD14-CD16+), show distinct functions in osteosarcoma. In primary osteosarcoma tissues, classical (CD14+D16-) monocytes with an overexpression of VCAN and S100A8/9/12 exhibit pro-inflammatory functions, whereas the non-classical (CD14-D16+) monocytes with high levels of *CDKN1C, LILRB2, TGAL*, and *CX3CR1* expression exhibit the anti-inflammatory effects (25).

The phenotypes of TAMs are linked to clinical outcomes in osteosarcoma. TAMs expressing CD14 or CD163 are associated with improved overall survival and metastasis-free survival in multiple osteosarcoma cohorts (25). However, the relationship between increased CD68+ TAMs and clinical prognosis in osteosarcoma patients is controversial. Elevated CD68+ TAMs are linked with either better overall survival or poorer (26) 5-year event-free survival (25). Interestingly, TAMs expressing both CCL18 and CD68 are correlated with lung metastasis and a worse prognosis (27).

TAMs in osteosarcoma consist of a heterogeneity of subpopulations, classified as anti-tumor M1-prolarized macrophages and pro-tumor M2-prolarized macrophages. TAMs infiltrate massively into osteosarcoma tissues and specific subpopulations are involved in a wide range of tumor progression pathways. Primary osteosarcoma tissues consist of high infiltration M2-prolarized TAMs. Liu et al. classified TAMs in treatment-naïve osteosarcoma based on the expression of *FABP5*, *NR4A3*, *TXNIP*, *IFIT1*, *MCM5*, *and MKI67* (25). They found that TXNIP+ TAMs exhibited M2 polarization with a high expression of M2 markers (*MERTK*, *MRC1*, *STAB1*, and *CD163*), whereas IFIT1+ TAMs displayed M1 polarization, regulated by STAT1 and characterized by an increased expression of IFN signaling and proinflammatory genes (*CCL2*, *CCL3*, *CCL4*, *CXCL9*, *CXCL10*, and *TNF*). M1-TAMs interacted with Tregs and exhausted CD8+ T cells through ligand receptors like LGALS9, PDCD1LG, CD274, and SP1. Zhou et al. also identified a high proportion of M2-like TAMs (*CD163*, *MRC1*, *MS4A4*, and *MAF*) in primary osteosarcoma patients receiving chemotherapy (28).

Hybrid TAM phenotypes also exist, indicating the plastic nature of TAMs in the TIME of osteosarcoma. Liu et al. found that NR4A3+ cells, identified as M2-TAMs in primary naïve osteosarcoma lesions, express both M1 and M2 phenotypes simultaneously (25). By inferring cellular trajectory, it was found that these NR4A3+ TAMs originate from FABP+ TAMs, forming a branched structure into M1 (IFIT1+ cluster) or M2-TAMs (NR4A3+ and TXNIP+ cluster). Lipid metabolism plays a role in regulating the M1/M2 polarization switch through multiple cellular pathways. The lipogenic phenotype of TAMs may serve as a metabolic hallmark influencing tumorigenesis and cancer progression in primary osteosarcoma. Correspondingly, genes associated with lipid metabolism are reported to correlate with the TIME and prognosis in osteosarcoma patients (29). The presence of immune cells with a high lipid metabolic profile is associated with poor prognoses in osteosarcoma patients (30).

#### 2.1.1 The roles of TAMs in chemoresistant and immunosuppressive mechanisms of osteosarcoma

**Figure 2:**
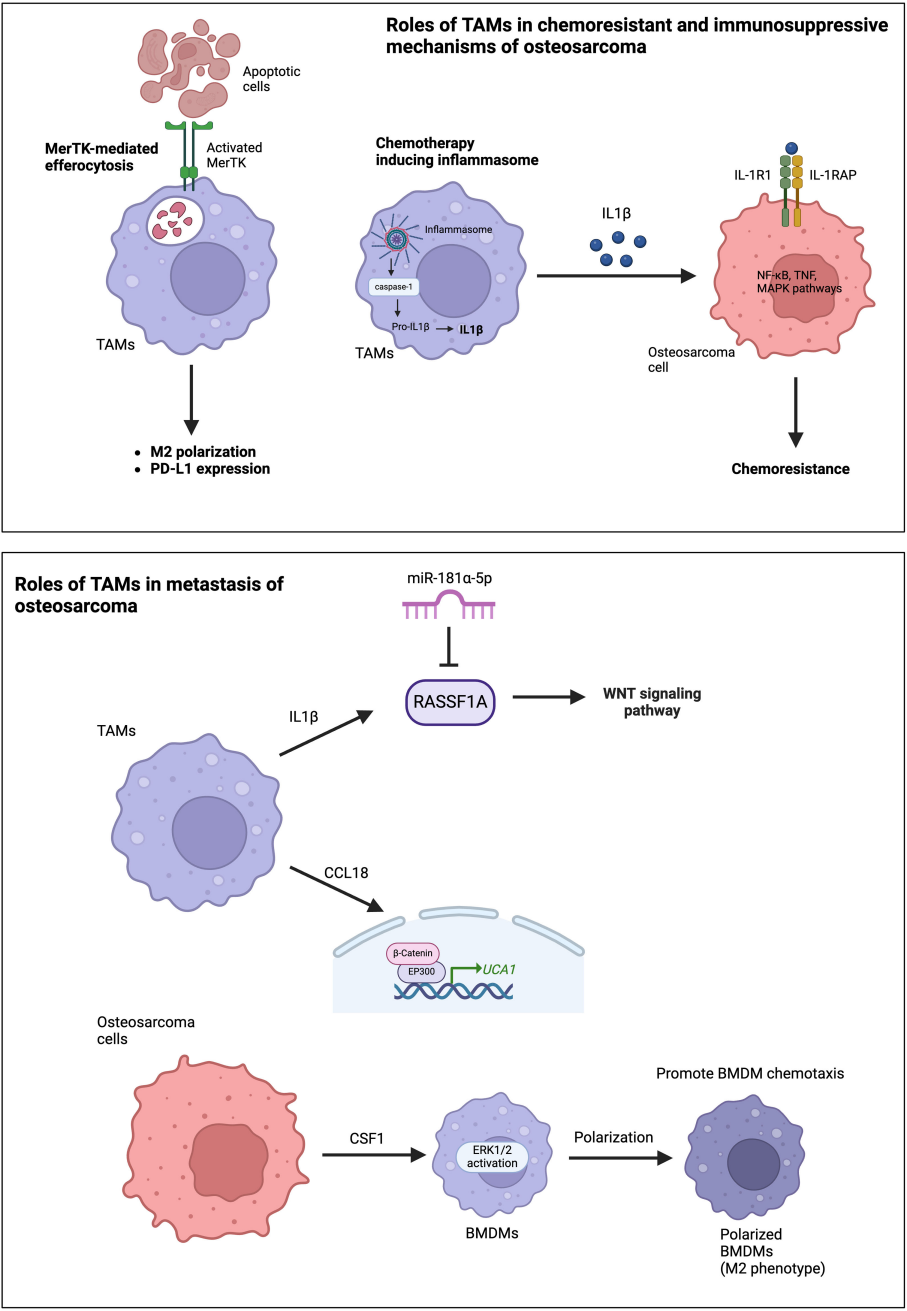
Roles of TAMs in chemoresistance, immunosuppressive mechanisms, and the metastasis of osteosarcoma.

Activated TAMs, particularly under neoadjuvant treatment, decrease the sensitivity of osteosarcoma cells to drugs by inhibiting tumor apoptosis and promoting cell survival (31). This effect is attributed to the secretion of IL1β by TAMs, which leads to the upregulation of IL1R1 and IL1RAP expression in osteosarcoma cells (**Figure 2**). IL1β treatment has been shown to reduce the sensitivity of osteosarcoma cells to chemotherapeutic agents in animal studies. Interestingly, IL1β secretion is triggered by the cascade signals from neoadjuvant treatment, promoting the assembly of inflammasomes in TAMs and activating the caspase pathway that induces the secretion of IL1β. Furthermore, TAMs are among the main immune cells expressing PD-L1 in the TME (32). The PD-1-PD-L1 signaling pathway is well-known for its impact on T-cell exhaustion and the reduction of T-cell function. ICIs, such as PD-1 or PD-L1 inhibitors, are the mainstay of immunotherapy in cancer treatment. The expression of PD-L1 and M2 polarization of TAMs are induced by MerTK-mediated efferocytosis, a critical macrophage function involved in clearing apoptotic bodies (**Figure 2**) (32). Blocking the MerTK-mediated efferocytosis pathway significantly suppresses osteosarcoma progression and immune tolerance. Therefore, inhibiting MerTK could be an effective approach to enhance osteosarcoma immunotherapy. Another immunosuppressive mechanism of TAMs involves the role of CD163+ M2-polarized TAMs in T cell exhaustion (33). CD163+ M2-polarized TAMs secrete immunosuppressive cytokines, such as IL-10 and TGF-β, which inhibit T-cell activation and proliferation, leading to T-cell exhaustion. Furthermore, CD163+ M2-polarized TAMs can express PD-L1, which induces an exhausted T-cell phenotype characterized by reduced cytokine production and proliferative capacity. Han et al. reported high levels of the exhausted T-cell subset in osteosarcoma, TIM-3+ PD-1+ T cells, correlated with the frequencies of CD163+ M2-polarized TAMs and tumor IL-10 concentration (33). Depletion of CD163+ M2-TAMs effectively increased T-cell proliferation and the production of proinflammatory cytokines.

The presence of CD163+ TAMs has been reported in various types of solid cancers with similar effects on the induction of T-cell exhaustion. In colorectal cancers, a high expression of CD163 on PD-L1 positive TAMs results in increased CD4+ lymphocyte infiltration, which contributes to upregulate PD-1 expression and the mediated PD-1/PD-L1 axis (34). In melanoma, CD163+ TAMs inhibited the recruitment of antitumor CD8+ T cells by suppressing the accumulation of Ly6C+, Nr4a1^neg^ monocytes (MNs) and CD11c^hi^ inflammatory TAMs (iTAMs). Upon depletion of CD163+ TAMs, there was a rapid mobilization of Ly6C+, Nr4a1^neg^ MNs, leading to an increased presence of CD11c^hi^ iTAMs. These iTAMs, in conjunction with CD4+ T cells, provoked the recruitment and activation of antitumor CD8+ T cells (35).

#### 2.1.2 The roles of TAMs in angiogenesis

Angiogenesis is a crucial mechanism enabling cancer cells to survive and metastasize to distant organs. In osteosarcoma, it has been observed that IL-34 can promote M2 macrophage polarization of osteosarcoma TAMs and induce angiogenesis (36). The association of angio-TAMs, characterized by high expression levels of angiogenic markers, has been identified in pre-chemotherapy biopsies from primary osteosarcoma lesions with and without metastasis (37). The frequency of angio-TAMs demonstrates a significant correlation with the malignant phenotype of osteosarcoma, with genes associated with angio-TAMs involved in biological processes linked to the malignant progression of tumors (38). Consistent with previous findings, the analysis of multiple datasets indicates that as the expression pattern of angiogenesis genes increases, the malignant degree of osteosarcoma also increases (39). Interestingly, a comprehensive quantification of the angiogenesis state may accurately differentiate prognosis, metastasis, and the therapeutic response for osteosarcoma patients.

#### 2.1.3 The roles of TAMs in the metastasis of osteosarcoma

The roles of TAMs in osteosarcoma metastasis primarily occur through the production and secretion of various cytokines and chemokines. The frequency of TAMs is higher in metastatic lesions than in corresponding primary lesions (40, 41). TAM-derived molecules, such as IL-1β and C-C motif chemokine ligand 18 (CCL18), significantly promote osteosarcoma metastasis (41). IL-1β secreted by M2-TAMs supports osteosarcoma metastasis via the RASSF1A-Wnt pathway (41). RASSF1A is a direct target of miR-181α-5p. Therefore, the RASSF1A-Wnt pathway could be targeted by miR-181α-5p and affected by nuclear factor-kappa B (NF-κB). CCL18 secreted from TAMs promotes osteosarcoma cell proliferation and migration via the EP300-UCA1-Wnt-β-catenin pathway (**Figure 2**) (27). CCL18 levels increased in the osteosarcoma tissues and serum of patients associated with lung metastasis. Furthermore, TAMs promote osteosarcoma cell metastasis through the stimulation of the epithelial-mesenchymal transition (EMT) of osteosarcoma cells via activation of the COX-2-STAT3 axis (40). The inhibition of COX-2 suppresses the metastasis of osteosarcoma cells in both *in vitro* and *in vivo* studies. Osteosarcoma cells also secrete colony-stimulating factor-1 (CSF1), which can stimulate ERK1/2 phosphorylation in bone marrow-derived macrophages (BMDMs), polarize BMDMs toward an M2 (TAM-like) phenotype, and promote BMDM chemotaxis (**Figure 2**) (42).

Osteosarcoma cells and TAMs communicate through the secretion of exosomes. Osteosarcoma cells increases the polarization of M2 TAMs, and exosomal miR-221-3p, secreted from M2-TAMs, further exacerbates the proliferation, migration, and invasion of osteosarcoma cells (43). Notably, SOCS3 is a target of miR-221-3p. The upregulation of miR-221-3p decreases SOC3 levels and activates the JAK2-STAT3 pathway. In addition, exosomes secreted from the osteosarcoma cell line can induce M2 polarizations of TAMs through the regulation of the expression of T-cell immunoglobulin and mucin domain (Tim) family proteins, particularly Tim-3, which promotes the migration, invasion, epithelial-mesenchymal transition (EMT), and lung metastasis of osteosarcoma cells (44).

### 2.2 Dendritic cells

DCs serve as efficient antigen-presenting cells and play a crucial role in orchestrating T-cell-mediated antitumor responses. DCs account for less than 5% of the total tumor-infiltrating myeloid cells in the TIME of osteosarcoma. A subcluster of DCs in osteosarcoma has been reported for their anti-tumor functions. Conventional DCs (cDCs) in primary osteosarcoma can be classified into two subsets: cDC1 (CLEC9A+ and XCR1+) and cDC2 (CD1c+, CLEC10A+, and FCER1A+) (25). Both subsets are widely recognized as key orchestrators of immune responses to cancer. Further characterization of four DC subclusters based on CD14/CD163, cDC1, cDC2, and CCR7 marker positivity demonstrates a higher number of cDC2 found in the TME of metastatic lung lesions than in primary and recurrent osteosarcoma (28). Furthermore, the CCR7+ DC subset specializes in directing DC mobilization to lymphoid organs and exhibits increased migratory speed, potentially indicating a close association with the metastatic potential of osteosarcoma (28). The chemokine receptor CCR7, expressed by cDCs, increases their migratory abilities from peripheral tissues to lymphoid organs, where these cells can elicit T-cell activation (45). Additionally, CCR7 employs distinct signaling pathways (such as PI3K-Akt, MAPKs, and RhoA) downstream to regulate the biased functionality of DCs, chemotaxis, controlling migratory speed, cytoarchitecture, and endocytosis (46). CCR7 and its ligand might be the key players that are closely related to metastatic sites and their axis regulates local anti-tumor activity as a means of controlling immune cell trafficking to tumors.

An analysis of a single-cell atlas of osteosarcoma and myeloid cells revealed that mature immunoregulatory dendritic cells (mregDCs), which are abundant in osteosarcoma samples, play a significant role in suppressing antitumor immunity in osteosarcoma (47). The mregDCs, expressing CCR7, LAMP3, and CD83, interact with Tregs through CD274-PDCD1 and PVR-TIGIT signaling, as well as their physical juxtaposition. The role of mregDCs in recruiting Treg cells, leading to an immunosuppressive microenvironment, has been observed in various cancer types (48). Studies in other cancers have demonstrated that mregDCs exert immunosuppressive functions by promoting the migration of Treg into the TME and interact with Treg through CCR4 binding and the CXCL9/10‐CXCR3 axis, among other mechanisms.

### 2.3 Tumor infiltrating lymphocytes

The heterogeneity of TILs clearly suggests their role in shaping and controlling interactive networks with other cells in the TIME. Phenotypic studies and the quantification of TIL subsets have demonstrated the immune system’s capacity, implying their involvement in modulating cancer progression and predicting responses to immunotherapies. scRNA-seq analysis of osteosarcoma tissues revealed that CD4-/CD8- (double negative; DN) T cells were one of the major types of lymphocytes, frequently observed in TILs of primary and advanced osteosarcoma tissues (25, 28). Cheng et al. also indicated that DN-TILs occupy the initial position in the trajectory plot and differentiate into CD4+ T cells and regulatory T cells (Tregs) (49).

#### 2.3.1 Effector T cells

Accumulative evidence emphasizes the predominant infiltration of exhausted CD8+ T cells in primary osteosarcoma tissues. Metastatic and recurrent osteosarcoma lesions exhibit a lower proportion of CD4+ and CD8+ TILs than primary lesions (28). In naïve primary osteosarcoma, the CD8+ T-cell subcluster is highly observed among tumor-TILs, further characterized based on the relative expression levels of cytotoxic-associated genes and regulatory factors. The C1_CD8+ subcluster represented naïve CD8+ T cells, displaying a high expression of JUND and FOSB, and a low expression of cytotoxicity genes (*GZMK*, *GZMA*, *GZMB*, and *PRF1*). The C2_CD8+ subcluster exhibited T-cell exhaustion signatures, expressing immune checkpoint-related genes (*PDCD1*, *CTLA4*, *LAG3*, *TIGIT*, and *HAVCR2*), CXCL13 chemokine, and tissue-resident genes (*ITGAE* and *ITGA1*). This dysfunctional subpopulation was also evident in advanced osteosarcoma lesions, marked by an elevated expression of T-cell exhausted inhibitory receptors (TIGIT and LAG3) (28). Finally, the C3_CD8+ subcluster represented cytotoxic T lymphocytes, characterized by the expression of CD69 and co-stimulatory genes, along with the TNF signaling pathways. Additionally, CD4+ TILs were uniquely observed by relatively high expression levels of cytotoxic GZMA and co-stimulatory molecules, including TNFRSF14, TNFRSF25, and ICOS5 (28). This concomitant expression suggests their ability to stimulate the cytotoxic activities of neighboring T cells.

#### 2.3.2 Regulatory T-cells

In the TIME, Tregs play a critical role in the evasion of immunological surveillance and reducing responses to immunotherapy. Intratumoral Tregs impair effector T-cell functions by producing the inhibitory cytokines IL-10 and IL-35, delivering bioactive TGF-β and inducing the apoptosis of effector T cells by depleting IL-2 via high-affinity IL-2Ra (CD25) (50). Indirect suppressive mechanisms involve the elimination of antigen-MHCII and CD80-CD86 through TCR and CTLA-4-mediated transendocytosis and trogocytosis events (50). The reverse signaling of CTLA-4 can induce the activation of the indoleamine 2,3-dioxygenase activity of APC to suppress the function of effector T cell (51). The study of the heterogeneity and immunosuppressive function of Tregs in naïve primary osteosarcoma demonstrated greater Treg infiltration than in normal bone, with a positive expression of *FOXP3*, *CD4*, *CTLA-4*, and *TIGIT* in Tregs (49). The abundant expression of *TIGIT* in Tregs is consistent with a previous study that identified *TIGIT* as widely present in various TIL subsets but most abundant in the Tregs of osteosarcoma (28). Interestingly, hallmark pathways involved in tumorigenesis and progression, such as oxidative phosphorylation, angiogenesis, and the mTORC1 pathway, were highly activated in Tregs from osteosarcoma tissues (49).

#### 2.3.3 B cells

Although only a very small proportion of B cells resided in the TIME, scRNA-seq analysis revealed unique heterogeneity in primary osteosarcoma lesions (25). Liu et al. explored five subsets of the B-cell population and a diversity of naïve, memory B cells, and plasma cells were observed. The naïve CD27- B cell was identified as follicular B cells expressing MS4A1 and CD79A/B, the phenotypic subtypes that mostly found lymphoid follicles of tertiary lymphoid structures (TLSs) of osteosarcoma tumor. Importantly, the naïve B-cell cluster also exhibited IGHD (IgD) and IGHM (IgM) characteristics. This unique phenotype has a migratory ability to undergo germinal centers. On the other hand, memory B-cell clusters that were identified as antibody secretory cells (expressing MZB1 and SDC1/CD138) showed an elevated relative expression of IGHG3 (IgG) but low IGHD and IGHM. The activated IgG memory B cells preferentially differentiate to a plasma cell fate. Additionally, plasma cells were identified with the high expression levels of immunoglobulin heavy chains, IGHG1, IGHG2, IGHA1 (IgA), and IGHA2. This subcluster was committed to mature plasma cells due to the expression of transcription factor PRDM1/Blimp1 (52).

#### 2.3.4 Natural killer cells

NK cells are a class of innate lymphoid cells that are recognized as non-specific cytotoxic immune cells. They possess the ability to control tumor growth and metastasis without requiring prior activation or sensitization (53). NK cells can eliminate cancer cells through complex mechanisms: releasing cytotoxic granules containing perforin, granzymes, and granulysin; generating cytokines (such as IFN-γ and TNF-α) to activate antitumor immunity; and death ligands, such as Fas ligand (FasL) and TNF-related apoptosis-inducing ligand (TRAIL) (54).

In osteosarcoma, NK cells were identified as a common TIL subset that could be classified into two subclusters based on their NK cell marker expression (NKG7 and GNLY) (28). One subcluster, expressing the T-cell markers CD3D and CD8A, was classified as NK T cells. These cells showed activation and a strong expression of *GZMB*, *GZMA*, and *IFN-γ*, indicating tumor cytotoxicity in osteosarcoma. The other subcluster, classified as NK cells, had only a small fraction expressing *GZMB*, *IFN-γ*, and *PRF1*, suggesting a non-activated state in osteosarcoma lesions (28).

### 2.4 Myeloid-derived suppressor cells

Myeloid-derived suppressor cells (MDSCs) are immune cells derived from the myeloid lineage that play a crucial role in the TME by suppressing the immune response and promoting tumor growth. MDSCs originate from bone marrow and consist of immature myeloid cells that fail to develop into mature cells, eventually differentiating into polymorphonuclear-MDSCs or monocytic-MDSCs. In osteosarcoma, MDSCs have been reported to heavily infiltrate the TME (55). The accumulated MDSCs within the TME suppress T-cell-mediated immune responses through the high expression of IL-18 and CXCL12 (55, 56). Furthermore, scRNA-seq analysis of six treatment-naïve osteosarcoma tumors, combined with a dataset of 22,035 cells from six osteosarcoma tumors, demonstrated that MDSCs were among the most abundant in the immunosuppressive milieu, as evidenced by MDSC hallmark genes (*VCAN*, *CLEC4E*, and *CSF3R*) (57).

### 2.5 Chondroblastic osteosarcoma cells

Chondroblastic cells have a valuable role in chondroblast-type osteosarcoma and are predominantly found in chondroid matrix production with variable cellularity. Four clusters of chondroblastic osteosarcoma derived from primary, recurrent, and lung metastasis were characterized based on the high expression levels of *ACAN*, *COL2A1*, and *SOX9* and their distinctive gene expression pattern (28). Among the chondroblastic osteosarcoma cells, the proliferating malignant chondroblastic osteosarcoma was identified by the expression of the gene-regulating tumor cell cycle, including *TOP2A*, *PCNA*, *TYMS*, and *MKI67*. On the other hand, two subclusters were hypertrophic chondroblastic cells that elevated the expression of the *MEF2C*, *PTH1R*, and *IHH* genes. Gene expression involving the IL-2-STAT5, Hedgehog, and Notch pathways was higher in heterotypic subcluster I, whereas the IL-6-JAK-STAT-mediated inflammatory pathway was highly expressed in heterotypic subcluster II. Finally, the last subcluster was described as trans-differentiated cells in which the osteoblastic differentiation genes, such as *RUNX2*, *SPP1*, and *COL1A*, were highly expressed. Gou et al. revealed two subclusters of chondroblastic cell populations, namely Osteosarcoma_3 and Osteosarcoma_8, for which the later subcluster was implicated in tumor invasiveness. Several genes associated with metastasis, such as *COL6A1*, *COL6A3*, and *MIF*, were found to be highly expressed, and gene enrichment analysis showed that the PI3K-AKT pathway was highly activated.

### 2.6 Osteoblastic osteosarcoma cells

Traditionally, osteosarcomas are mostly derived from osteoblasts. It is well known that osteoblasts participate in new bone formation during the bone remodeling process. Many studies demonstrated that osteoblastic cells are shown to be important players in the development of osteosarcoma. The comprehensively analyzed single-cell dataset of treatment-naïve osteosarcoma represented cancer cell subpopulations based on their divergent phenotypes in primary tissues (25, 58). Liu et al. investigated five osteoblastic osteosarcoma clusters (C1-C5) that differentially expressed the genes corresponding to multifaceted physiological traits, including inflammatory markers, cell-cycle proliferation, cell metabolism associated with carbohydrate transmembrane transporter activity and glucose catabolic processes, extracellular matrix regulation, and ossification (25) Remarkably, osteoblastic-C1 and C5 displayed the most malignant stage by showing an increased expression of genes associated with a poor prognosis. With the same single-cell dataset, Zeng et al. found that the specific clusters of osteogenic cancer stem cell (CSC)-like tumor cells had a chemoresistant-related expression profile annotated by bulk RNA results (58). These subclusters bridged between tumor and non-tumor cells by stimulating several growth factors to promote themselves into a proliferative stage. For osteosarcoma patients receiving chemotherapy, the transcriptional heterogeneity of malignant osteosarcoma cells showed six subclusters belonging to osteoblastic lineages in primary, recurrent, and lung metastatic lesions (28). Osteoblastic-C1 and -C2 typically expressed proliferation markers with the cell cycle-regulated transcripts of S phase genes (C1: *PCNA*, *TYMS*, and *RRM*2) and G2/M phase genes (C2: *UBE2C* and *HMGB2*). Osteoblastic-C3 was functional in angiogenesis and the IFN-α and IFN-γ signaling pathways, whereas C4 was involved in MYC and oxidative phosphorylation. Osteoblastic-C5, enriched in the TGF-β, P53, KRAS, and hypoxia pathways, and C6 displayed a significant increase of myogenesis and inflammatory responses as well as immune rejection signaling pathways (58). These subclusters bridged between tumor and non-tumor cells by stimulating several growth factors to promote themselves into a proliferative stage.

Transcriptomic profiling of osteoblastic osteosarcoma cells demonstrated a higher activation of oxidative phosphorylation, reactive oxygen species, mTORC1, hypoxia signaling pathways, and MYC gene targets in lung metastases than primary and recurrent tissues. Likewise, the hypoxia, TNF-α, TGF-β, IL2-STAT5, and mTORC1 pathways were functionally enriched in recurrent lesions. These signaling pathways may contribute to osteosarcoma chemotherapeutic resistance and tumor relapse.

### 2.7 Osteoclasts

In addition to osteoblasts, osteoclasts play a crucial role in the pathogenesis of osteosarcoma by mediated osteolysis. Osteoclasts are unique multinucleated cells responsible for the resorption of bone during bone homeostasis (59). Osteoclasts dysfunction is associated with osteosarcoma pathology by which their elevated osteoclast activity contributes to sustained proliferation and survival (60). Through scRNA-seq analysis, four clusters of osteoclasts were dissected in naïve primary osteosarcoma tissues, where progenitor and mature cells were two major subclusters and hypofunctional and non-functional osteoclasts were minor subclasses (25). According to the specific gene expression, myeloid markers (*CD74*, *CD14*, *HLA-DRA*, and *MKI67*) were highly expressed in progenitor cells but decreased in mature osteoclasts. Trajectory analysis showed that the minor osteoclast subpopulations were located at a terminal position in pseudo-time where the expression levels of osteoclast markers were decreased. Cellular interaction analysis suggested that the differentiation of osteoclasts was regulated by osteoblastic cells through the TNFSF11-TNFRSF11A interaction. Osteoclasts have been classified into three major types of progenitor, immature, and mature osteoclasts in advanced osteosarcoma (28). Progenitor osteoclasts could differentiate into mature osteoclasts and exhibited a hyperproliferative phenotype due to the high expression of TOP2A. Immature osteoclasts were positive for osteoclast and myeloid markers, whereas mature osteoclasts had high osteoclast marker expression levels compared with progenitor cells. The distribution of osteoclast clusters was respective to the progressive stage of osteosarcoma. Interestingly, osteoclast infiltrations were relatively lower in lung metastases and recurrent lesions than in primary lesions. The tissue-specific accumulation suggested a significant burden of osteoclasts in the TIME of advanced osteosarcoma.

### 2.8 Tumor-associated stromal cells

#### 2.8.1 Stromal mesenchymal stem cell

The diversity of MSC populations were identified based on the markers CD10, CD90, and CXCL12 by Zhou et al. Clustering analysis revealed three MSC subsets through the differential expression of feature genes involved in metastasis and the following mesenchymal progenitors: NT5E+-MSCs, WISP2+ -MSC, and CLEC11A+-MSC clusters. NT5E+-MSCs was shown to stimulate angiogenesis and metastasis, whereas WISP2+-MSCs and CLEC11A+-MSCs were associated with the promotion of metastasis and differentiation of mesenchymal progenitors into mature osteoblasts, respectively (28). Systematically cell mapping by scRNA-seq analysis revealed the stem-like population in chemotherapy-resistant osteosarcoma having stem cell marker CD117, MYC oncogene, epigenetic regulator JMJD3, and angiogenesis marker VEGFR2. Interestingly, the JMJD3+/VEGFR2+ subset concomitantly expressed stem cell markers CD117, indicating its stem cell quiescence. By using pseudo-time ordering analysis, it was shown that the JMJD3-VEGFR2 positive subset expressing CD117 potentially differentiated toward chondrocyte-like or fibroblast-like cell lineages. This suggested the stem-like/progenitor cells were involved in the hierarchy of therapy-resistant osteosarcoma. Based on this evidence, immunofluorescence staining of chemo-resistant osteosarcoma lesions was further confirmed by the high level of JMJD3+/VEGFR2+, and double positive cells were observed in the those tissue samples (61). The inhibition of VEGFR2 and JMJD3 synergistically impeded osteosarcoma cell propagation and tumor growth.

#### 2.8.2 Cancer-associated fibroblasts

Within the TIME of treatment-naïve osteosarcoma tissue samples, CAFs contribute to the malignant phenotype of osteosarcoma cells by stimulating the proliferation and invasion of osteosarcoma cells (25). Based on the expression of the common CAF gene signature, CAFs formed the distinct subclusters by which each of them were involved in (1) tumor angiogenesis and invasion (MMP9 and MCAM) (2), osteoblast proliferation development and ossification, and (3) cell cycle and cell proliferation. Notably, these CAFs exhibited heterogeneous gene expression promoting angiogenetic behavior through ligand-receptor mediating angiogenic signaling pathways. scRNA-seq analysis by Zhou et al. also found diverse CAF clusters isolated from advanced osteosarcoma lesions, which showed remarkably high levels of the fibroblast markers decorin (DCN) and lumican (LUM) (28). CAF clusters were sub-categorized into (1) COL14A1+ ACTA2+ matrix fibroblasts, (2) smooth muscle-like fibroblasts (increased expression of DES along with the downregulation of ACTA2 and COL14A1), and (3) a myofibroblast cluster (a high level of MYL9, LUM, and ACTA2 expression alongside no expression of COL14A1 and DES) that exhibited strong osteoblast marker expression (IBSP and SPP1), suggesting their function as an osteoblast-like phenotype. These findings implied that most CAF subpopulations were seemingly dysfunctional in advanced osteosarcoma, where they lack the common functionality genes inherited in the fibroblast. COL14A1+ matrix fibroblasts and myofibroblast phenotypes were predominantly found in primary and recurrent osteosarcomas, whereas smooth muscle-like fibroblasts were foremost in metastatic lesions. Previously, it was noted that the expression of ACTA2 was associated with distant metastasis in lung adenocarcinoma (62) and the clinical response to the ICI of gastric cancer patients (63). Therefore, ACTA2 expression in CAF might be a favorable target for osteosarcoma management as a prognostic biomarker and/or therapeutic target. Furthermore, the scRNA-seq datasets of naïve (GSE162454) and advanced (GSE152048) osteosarcomas were compared by Huang et al. Across the different tissue sample types, CAF populations of recurrent lesions had a higher infiltration level than primary and lung metastatic samples. In this respect, the pathway enrichment analysis revealed that the EMT pathway was increasingly activated in the particular CAFs, with a high expression level of lysyl oxidase (*LOX*) genes. *LOX* expressed by CAFs was associated with immune infiltration levels and EMT state, which in turn contributed to a poor prognosis. Further experiments demonstrated that the upregulated *LOX* promoted tumor progression, metastasis, and poor overall survival in different tumors (64–66). Therefore, the reconstructed analysis suggested *LOX* as a promising therapeutic target for recurrent osteosarcoma.

## 3 Cell-cell communication in the TIME of osteosarcoma

**Figure 3:**
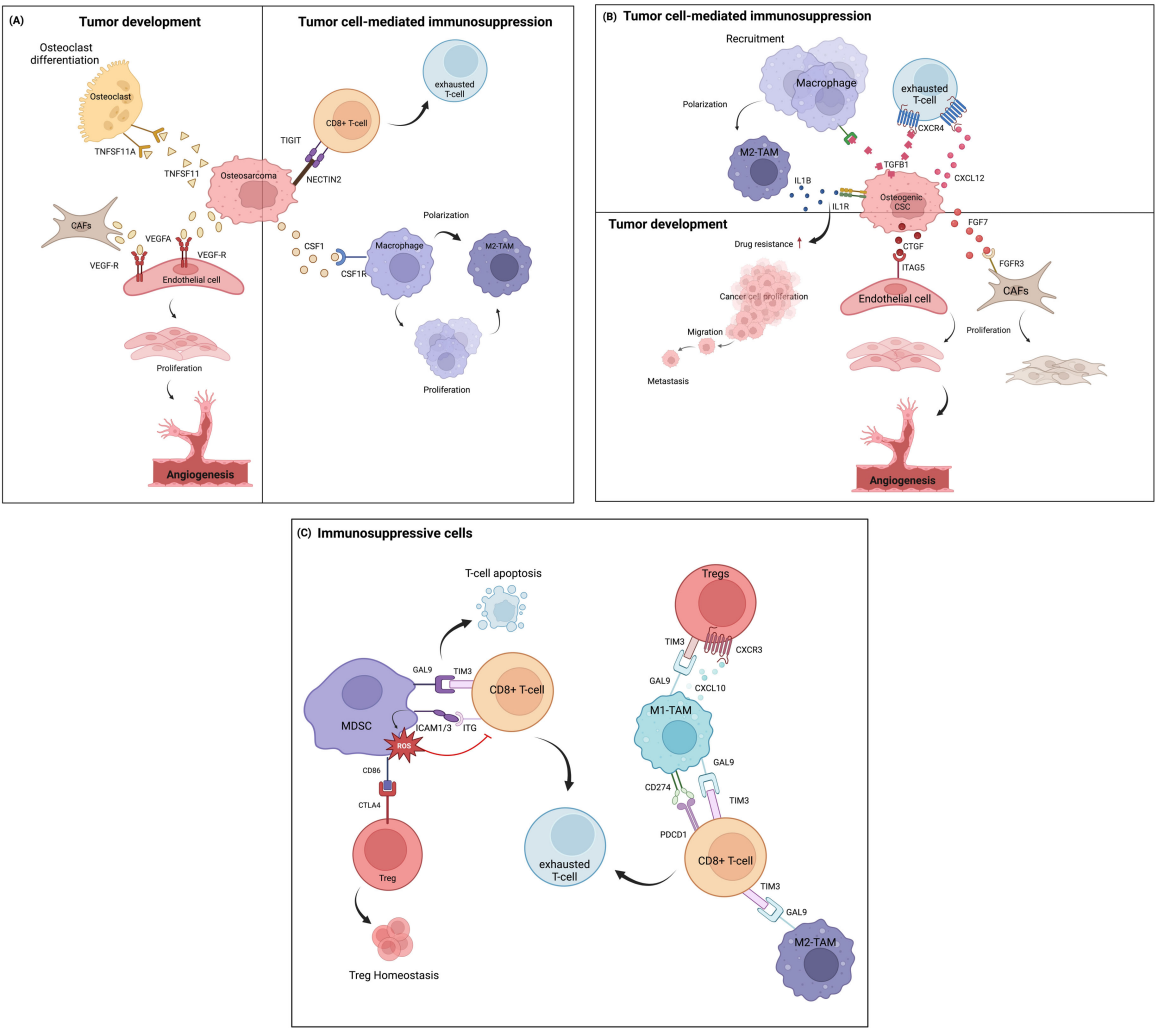
Cell-cell communication in the TIME of osteosarcoma, based on ligand-receptor interactions at the single-cell level. **(A)** Communication between osteosarcoma cells and other immune cells and other cell types in the TME. **(B)** Intercellular communication among osteogenic CSCs and other cells. **(C)** Communication involving immunosuppressive cells.

Within the complex environment of osteosarcoma, intercellular communication among different cell types plays significant roles in tumor development and immunosuprression. During osteosarcoma development, osteosarcoma cells directly influence osteoclasts by secreting various signaling molecules that shift osteoclast activity toward disrupting bone, thereby promoting the onset of osteosarcoma (**Figure 3A**). These mechanisms include the activation of osteoclast through TNFSF11-TNFRSF11A interaction triggered by TNSF1 secreted from osteoblastic osteosarcoma clusters (25, 67). TNFSF functions in the bone induce osteolysis and the differentiation of progenitor cells into mature osteoclasts. Additionally, the interaction of VEGFA produced by osteoblastic osteosarcoma cells and CAFs can stimulate endothelial cell proliferation through VEGF receptor (VEGFR) binding, leading to a new vascular formation that supports osteosarcoma cell survival (68).

Osteosarcoma cells also play important roles in immunosuppression within the TME, mainly through the inhibition of T-cell functions and the induction of macrophage polarizarion. In this context, the immunoregulatory NECTIN2-TIGIT interaction between osteosarcoma and CD8+ T cells induces TIGIT-mediated T-cell suppression by impairing CD8+ T-cell proliferation and activation (69). This interaction possibly exerted immunosuppressive activity by blocking co-stimulatory signaling via the counterpart CD226, which typically modulates anti-tumor immunity and inflammatory responses (70). Additionally, a paracrine loop of CSF1 secreted from osteosarcoma cells in the tumor niche continuously activates M2-TAM, leading to the differentiation and polarization of macrophages (71, 72), as well as aggravates tumorigenesis (73).

Other important cells known for their chemoresistant phenotypes, osteogenic CSCs, exhibit significant roles in intercellular communication within the TIME of chemo-naïve osteosarcoma tissues (**Figure 3B**) (58). Osteogenic CSC clusters release growth factors (FGF and CTGF) that induce CAF growth and vascular endothelial formation by binding to their receptors. CAF clusters can express various cytokines and factors that stimulate vascularization and trigger angiogenesis (74). These signal transductions further induce therapeutic resistance and tumor progression (75, 76). TGFβ-1 secreted by osteogenic CSCs is involved in the regulation of immune cell function through TGFβ signaling effects by inducing monocyte recruitment and activating them into the M2-TAM state (77). Moreover, TGF-β and CXCL12 often orchestrate cancer progression by depleting the T-cell response through the TGFβ1-CXCR4 or CXCL12-CXCR4 axes (78, 79). Osteogenic CSCs also facilitate TAM-mediated IL-1β production. IL-1β release can lead to the transcription of signaling pathways previously found to promote chemoresistance in osteosarcoma (31) and initiate a pro-tumoral response contributing to metastasis (80, 81). These M2-TAM populations, in turn, definitely support tumor progression.

Apart from the tumor cells, the TIME of osteosarcoma presents a wide range of immune cell types that largely contribute to immunosuppressive milieus (**Figure 3C**). Myeloid cells constitute the highest proportion of cells in tumor tissues. MDSCs play an important role in cellular network regulation by suppressing T-cell-mediated immune responses, which are relevant to the clinical outcome of cancer (82). MDSC signaling to exhausted T cells and Tregs has been investigated in the TIME of osteosarcoma (83). It was shown that the strong interaction between the GAL9 ligand and its receptor TIM3 on T cells promotes the apoptosis of CD4+ and CD8+ TILs (84, 85). Similarly, highly expressed ICAM1/3 in MDSCs, which bind with ITG receptors (ITGB2, IL2RG, and ITGAL) expressed on T cells, contributes to the ROS-dependent inhibition of T-cell activation. This suppressive activity depends on CD11b-dependent physical contact via cell-cell contact-dependent mechanisms (86, 87). MDSCs have also been shown to indirectly suppress T-cell activation by inducing regulatory T cells. In the osteosarcoma TIME, this occurs through the interaction of CD86 molecules on MDSCs, which serve as ligands for CTLA4 on Tregs. The CTLA4-CD80/CD86 signal is a well-known ICI pathway, in which high-affinity binding limits the further activation of effector T cells, thereby maintaining the suppressive function of Tregs. Previous studies reported that CTLA4-CD80/CD86 signaling maintains homeostatic proliferation and a regulatory phenotype of Tregs, and anti-CTLA4 blockade treatment provides a reversible effect (88, 89).

On the one hand, ligand-receptor interactions were also identified between TAMs and subsets of T cells. Both M1- and M2-TAM phenotypes participate in T-cell inhibitory signaling by regulating Tregs and inducing CD8+ T-cell exhaustion through chemokine (CXCL9-CXCL10 signaling through CXCR3) and T-cell immune checkpoint (GAL9-TIM3 and CD274-PDCD1 signaling) pathways. These signaling pathways are crucial suppressors of the cytotoxic immune response. Blockade of the GAL9-TIM3 and CD274-PDCD1 pathways reinvigorates exhausted T cells and has shown favorable therapeutic efficacy in various malignancies (90–93).

## 4 Mechanisms modulating the tumor-immune microenvironment in osteosarcoma

In recent years, there has been growing focus on the TIME as a potential therapeutic target in osteosarcoma. Understanding the mechanisms influencing the TIME in osteosarcoma (**Table 1**) is crucial for comprehending tumorigenesis, evolution, progression, and metastasis. This knowledge provides valuable insights for developing novel therapeutic approaches, including molecularly targeted therapies and innovative immuno-oncology strategies, by elucidating the mechanisms that modulate the TIME in osteosarcoma.

**Table 1:** Studies of the alteration of the genomic and epigenetic modulation of the tumor-immune microenvironment in osteosarcoma.

Genomic alterations			
Study	Population	Findings
Pires, S.F et al. (94)	• 28 Brazilian treatment-naïve osteosarcoma individuals •	• 445 potentially deleterious SNVs/indels and 1,176 copy number alterations (CNAs). *TP53* was the most frequently altered gene. • A protein-protein network enrichment revealed biological pathways associated with immune response and bone development.
Wu, CC et al. (95)	• 48 pediatric and adult patients with high-grade osteosarcoma •	• The median immune infiltrate level in high-grade osteosarcoma with poor-risk and adverse survival outcome was lower than other cancer types, with concomitant low T-cell receptor clonalities. • High immune infiltrate represents an enrichment of tumor-intrinsic immunosuppressive pathways • Low immune infiltrate showed a high number of deleted genes and negatively correlated with *PARP2* expression levels
Xie, L et al. (96)	• 12 high-grade osteosarcoma patients with initial bone metastasis and 26 patients with initial pulmonary metastasis	• Initial bone metastasis group carried more single-nucleotide variations. • Initial pulmonary metastasis exhibited structural variants. • Initial bone metastasis group exhibited better immunogenicity in the tumor microenvironment.
Liu, R et al. (97)	• Normalized sequencing datasets of osteosarcoma from Gene Expression Omnibus (GEO), GSE126209 • 11 expression datasets of osteosarcoma tissues and the 11 datasets of normal adjacent tissues	• Osteosarcoma disease-related immune cell populations, mainly Mast cells activated were enriched in osteosarcoma tissue. • Nine genes with varying levels of immune cell infiltration were associated with osteosarcoma, four of which, including *SORBS2*, *BAIAP2L2*, *SNAPC3*, and *ZDHHC21*, had a greater disease-free survival probability than the high abundance group.
Epigenetic alterations			
Study	Population	Findings
Mills, LJ et al. (98)	• 24 treatment-naïve osteosarcoma individuals	• Low abundance of stromal and immune cells in human osteosarcoma samples were predicted by a custom signature file for CIBERSORT. • Most methylation clusters showed positive correlations with mesenchymal stromal cells and were less influenced by immune cell abundance.
Shi, D et al. (99)	• Multi-omics data for osteosarcoma patients from the Therapeutically Applicable Research to Generate Effective Treatments (TARGET) and Gene Expression Omnibus (GEO) databases	• Three immune methylation patterns (IMPs) of osteosarcoma patients were cluster based on methylation levels of CpG sites related to immunologic gene sets. • Six gene signatures (*MYC*, *COL13A1*, *UHRF2*, *MT1A*, *ACTB*, and *GBP1*) were constructed to predict osteosarcoma prognosis. • Osteosarcoma patients in the high-IMP Risk group had higher infiltrations of potential immunosuppressive cells, higher infiltrations of naïve CD4 + T cells, and lower infiltrations of activated NK cells, potentially leading to an immunosuppressive TME status and a poor response to ICI therapy.

### 4.1 Mutations

The point mutation burden of osteosarcoma is approximately 1.5 per Mb (100), giving it the greatest mutation burden among pediatric solid tumors but intermediate overall and much lower than other type of cancers such as melanoma or non-small cell lung cancer. Recent investigations involving whole-genome sequencing (WGS) and molecular profiling in osteosarcoma have revealed substantial occurrences of structural changes in chromosomes, such as rearrangements due to chromothripsis (ranging from 20% to 89%), along with the presence of mutation clusters termed kataegis (found in 50% to 85% of cases). These phenomena contribute significantly to osteosarcoma heterogeneity but are associated with limited recurrent alterations that can be clinically targeted (100–102). The association of osteosarcoma mutational profiles and the TIME includes clusters of immune cells ranging from low to high levels of immune infiltrate. High immune infiltrate is associated with an enrichment of tumor-intrinsic immunosuppressive pathways, indicating the increased expression of signals that inhibit T-cell activation (PD-L1, CTLA4, and IFN-γ) and the IDO1 molecule involved in immunosuppressive cell recruitment (95). Conversely, low immune infiltrate is associated with a greater number of deleted genes, *TP53* being among the top-hit loss genes (95). This observation aligns with recent studies indicating that high levels of genome aneuploidy in cancer are associated with lower levels of immune-related markers (103, 104). Furthermore, the study demonstrates a significant negative correlation between the expression levels of poly(ADP-ribose) polymerase 2 (*PARP2)* and immune infiltrate in osteosarcoma samples (95).

### 4.2 Epigenetics

Hypermethylation in promoter regions can epigenetically silence tumor suppressor genes during oncogenesis. Simultaneously, abnormal DNA methylation in non-promoter regions significantly contributes to intratumoral diversity. Deyao S et al. identified three immune methylation patterns (IMPs) that can be used to construct a signature scoring model based on six genes (*MYC*, *COL13A1*, *UHRF2*, *MT1A*, *ACTB*, and *GBP1*) to predict osteosarcoma prognosis (99). High-IMP_Risk patients exhibited aggressive features, with activated MYC targets and tumorigenesis-related pathways, whereas low-IMP_Risk patients showed intense immune responses. High-IMP_Risk patients might have a stronger immunosuppressive microenvironment, potentially limiting the efficacy of immunotherapy. Additionally, high-IMP_Risk patients displayed genetic amplifications in oncogenes, including MYC and MCL1, that might be potential therapeutic targets for osteosarcoma treatment.

N6-methyladenosine (m6A) is a prevalent RNA modification crucial for regulating gene expression. Dysregulation of m6A, often observed in cancer, can alter mRNA stability, splicing, and translation, leading to oncogenic changes in gene expression patterns (105). In osteosarcoma, m6A plays multifaceted roles in the TME. It affects metabolic dysregulation by regulating glycolysis, influencing glucose uptake, lactate production, and ATP levels through interactions with circ-CTNNB1 and RBM15 (106).

Additionally, m6A-associated non-coding RNAs (ncRNAs) influence the TIME in osteosarcoma, affecting various immune cell populations that potentially impact tumor initiation and progression (107, 108). Yikang et al. demonstrated that TNS1 antisense RNA 1 (TNS1-AS1) and TFPI2 divergent transcript (TFPI2-DT) expressions were positively correlated with the levels of memory B cells and naïve B cells in osteosarcoma. Their findings also revealed a correlation between the expression of various long non-coding RNAs (lncRNAs) and the levels of immune cells that might be involved with the immunosuppressive microenvironment. Their findings also revealed a correlation between the expression of various long lnRNAs and the levels of immune cells that might be involved with the immunosuppressive microenvironment. For instance, LINC00910 expression showed a negative association with CD8+ T cells, and LINC00538 had a positive correlation with resting dendritic cells but a negative correlation with activated dendritic cells (107). Furthermore, the m6A-related lncRNAs had prognostic significance: lncRNAs in cluster 1 were associated with lower survival rates than those in cluster 2, which was notably enriched with immune plasma cells (108).

### 4.3 Extracellular vesicles

Extracellular vesicles (EVs), released by tumor cells, are membranous structures containing proteins, nucleic acids, and other biomolecules. These EVs facilitate intercellular communication within the TIME and with distant cells. Their role in cancer progression involves the modulation of cellular processes, the promotion of angiogenesis, immune evasion, and the creation of a supportive milieu conductive to tumor advancement (109–111). In osteosarcoma, EVs play a crucial role in reprogramming various cell types, especially MSCs, within the TIME (112). The osteosarcoma-derived EVs increase the angiogenic activities of endothelial cells, induce macrophage dedifferentiation, and increase the number of osteoclast-like cells and CAFs in both local and metastatic sites (113, 114). Furthermore, these EVs can induce a tumor-like phenotype in non-transformed cells, indicating their involvement in oncogenic transformation (115). Osteosarcoma EVs have been shown to promote epigenetic changes in MSCs, which are highly susceptible to EV-mediated transformation (112). As MSCs are considered potential cells of origin for osteosarcoma (116), their reprogramming by osteosarcoma EVs might be an early event in osteosarcoma development. Baglio et al. elucidated that membrane-associated TGF-β was highly observed in exosomes derived from highly metastatic osteosarcoma. Once internalized by MSCs, it induced the proinflammatory cytokine IL-6, thereby promoting pro-metastatic and pro-tumorigenic phenotypes *in vivo* (117). This finding highlights the significant role of exosomal proteins in the development and progression of osteosarcoma.

Tumor EVs carrying tumor-specific antigens (TSAs) and major histocompatibility complex (MHC) can act as decoys or directly activate T cells, with improved antigen presentation when interacting with mature dendritic cells (DCs). This underscores the complex mechanisms employed by EVs in immune evasion and modulation within the osteosarcoma microenvironment. The downregulation of MHC molecules and TSAs poses a significant challenge in osteosarcoma treatment by hindering the immune system’s ability to recognize and target cancer cells (118–120). Tumor EVs carrying TSAs serve as decoys, redirecting anti-tumor immunity away from cancer cells. These EVs can be taken up by immune and non-immune cells, potentially disrupting the immune response. Interestingly, EVs containing TSA-MHC complexes can directly activate T cells, and their effectiveness in antigen presentation is significantly increased when attached to the surface of mature DCs (119, 120).

## 5 Therapeutic perspectives

A deepening understanding of the biological characteristics of osteosarcoma and rapid advancements in understanding the osteosarcoma-TIME have accelerated the development of immunotherapies. Promising monotherapy or combination approaches involving tumor vaccines, ICIs, immunomodulators, and genetically modified T cells, aim to increase treatment efficacy while minimizing side effects, offering hope for improved outcomes. The immune cocktail therapy, which combines various immunotherapeutic strategies, has shown promise in modulating the cancer-immunity cycle for more effective osteosarcoma treatment. We summarize the ongoing or planned clinical experiments exploring these strategies in **Table 2**.

**Table 2:** Ongoing or planned clinical trials of immunotherapies for osteosarcoma.

Approach	Clinical settings	Phase	NCT number	Status
PD-1 inhibitor + CTLA-4 inhibitor	Recurrent/refractory	I+II	NCT02304458	Completed
HER-2 inhibitor + chemotherapy	Newly diagnosed/recurrent	II	NCT04616560	Suspended
B7-H3 CAR T cells	Recurrent/​refractory solid tumors including osteosarcoma	I	NCT04483778	Active, not recruiting
GD2 CAR-modified VZV-specific T cells + fludarabine+ cyclophosphamide	Recurrent/refractory	I	NCT01953900	Active, not recruiting
TCRαβ+/CD19+ depleted haploidentical HSCT + zoledronate	Relapsed with pulmonary or bone metastases	I	NCT02508038	Recruiting
TIL T cells + PD-1 inhibitor + CTLA-4 inhibitor	Recurrent/refractory	II	NCT03449108	Active, not recruiting

Although immunotherapy holds potential for treating osteosarcoma, its efficacy is currently limited by insufficient T-cell infiltration and an immunosuppressive TME (121). Oncolytic viruses have demonstrated potential in overcoming resistance to PD-1 blockade by increasing CD8+ T-cell infiltration (122). Additionally, angiotensin inhibitors have been proposed to mitigate extracellular matrix sclerosis, thereby improving tumor responsiveness to checkpoint immunotherapy in solid tumors (123). Biodegradable nanoparticles may function as adjuvants, targeting specific sites and eliciting inflammatory chemokines within the TIME, consequently promoting T-cell infiltration (124, 125).

## 6 Conclusion

Overall, the modulation of the TIME in osteosarcoma involves a complex interplay between genomic, immunologic, and epigenetic mechanisms, as well as intercellular communication among different cell types in the TME. Understanding these intricacies at the single-cell level and within the spatial landscape of the TME is crucial for developing novel therapeutic strategies, including targeted therapies and immunotherapies, to improve outcomes in osteosarcoma treatment.
